# Ultra-Processed Foods as Ingredients of Culinary Recipes Shared on Popular Brazilian YouTube Cooking Channels

**DOI:** 10.3390/nu14183689

**Published:** 2022-09-07

**Authors:** Anice Milbratz de Camargo, Alyne Michelle Botelho, Állan Milbratz de Camargo, Moira Dean, Giovanna Medeiros Rataichesck Fiates

**Affiliations:** 1 Nutrition in Foodservice Research Centre, Nutrition Post Graduation Program, Universidade Federal de Santa Catarina, Campus Universitário João David Ferreira Lima—Trindade, Florianópolis 88040-900, SC, Brazil; 2 Computer Science Undergraduate Course, Universidade Regional de Blumenau, Rua Antônio da Veiga, 140, Blumenau 89012-900, SC, Brazil; 3 Institute for Global Food Security, School of Biological Sciences, Queen’s University Belfast, Belfast BT9 5DL, UK

**Keywords:** social media, social network site, Internet, cookery channels, recipe quality, cooking instruction, ultra-processed foods

## Abstract

Social media platforms are readily accessible sources of information about cooking, an activity deemed crucial for the improvement of a population’s diet. Previous research focused on the healthiness of the content shared on websites and blogs, but not on social media such as YouTube^®^. This paper analysed the healthiness of 823 culinary recipes retrieved from 755 videos shared during a six-month period on ten popular Brazilian YouTube^®^ cooking channels. Recipes were categorized by type of preparation. To assess recipes’ healthiness, ingredients were classified according to the extension and purpose of industrial processing, in order to identify the use of ultra-processed foods. Additionally, a validated framework developed from criteria established in both editions of the Dietary Guidelines for the Brazilian Population was employed. Recipes for cakes and baked goods, puddings, snacks and homemade fast foods, which were among the most frequently posted, contained the lowest proportion of unprocessed/minimally processed ingredients and the highest proportion of ultra-processed ingredients. Recipes containing whole cereals, fruits, legumes, nuts, and seeds were scarce. Results indicate that users should be critical about the quality of recipes shared on YouTube^®^ videos, also indicating a need for strategies aimed at informing individuals on how to choose healthier recipes or adapt them to become healthier.

## 1. Introduction

Public health initiatives from many countries encourage home cooking as a health-promoting strategy [1]. This is also true for both editions of the Dietary Guidelines for the Brazilian Population [2,3], which adopt distinct but complementary approaches for the promotion of healthy eating. The first edition of the Guidelines valued the act of eating at home and provided information on how to prepare food in a healthy way. Its directives were based on the intake of adequate amounts of foods, classified into food groups, to prevent nutritional deficiencies and chronic non-communicable diseases [2].

Aside from stressing the importance of home cooking, the Dietary Guidelines for the Brazilian Population published in 2015 focused on categorizing foods according to the extension and purpose of industrial processing [3]. Individuals should base their diets on unprocessed/minimally-processed foods (U/MP) and avoid ingesting ultra-processed foods (UP) as much as possible [3]. UP foods are formulations of ingredients that are usually nutritionally unbalanced, being rich in fats and sugars while poor in fibre and micronutrients [3]. Carbonated soft drinks, packaged snacks, mass-produced breads, margarines, candies, cake mixes, and many ready-to-heat frozen products (pies, pizza, sausages, burgers) are examples of UP foods [3,4].

High consumption of UP foods has been associated with chronic non-communicable diseases and all-cause mortality [5,6,7]. Conversely, cooking at home more often has been associated with a lower risk of developing chronic non-communicable diseases [8,9], possibly as a result of a better diet quality [9,10] due to the use of fresh ingredients. A pattern of healthy cooking practices, where individuals can confidently cook several meals using fresh foods and natural seasonings, and use healthier cooking techniques, was inversely associated with ultra-processed food consumption [11]. A diet composed mostly of U/MP foods, however, can only be achieved if individuals master a certain number of cooking skills [3].

Informal cooking education happens through culinary socialization over the course of a person’s life, a process in which individuals acquire patterns of practices and perceptions related to cooking, from socializing agents [12]. The first culinary socializing agents are family members; later in life, different agents start to influence cooking practices, such as friends, partners, cookbooks, culinary television programs, and more recently, the Internet [9,13,14,15]. Individuals report favouring Internet searches and digital sources when looking for recipes, instead of printed sources such as books, for the convenience of being ‘at hand’ [13].

Brazilians spend an average of 3.5 h daily on the Internet [16], mainly accessing social media [17]. Social media platforms have become accessible sources of information regarding cooking-related matters—people use Facebook^®^, Instagram^®^, Pinterest^®^ and YouTube^®^ to share and search for recipes, and to find meal suggestions and inspiration [13,18,19,20].

YouTube^®^ was created in 2005 and works as a video sharing platform, which is accessible via personal computers or smartphones through an Internet browser or application [21]. On the platform’s homepage, an algorithm suggests videos based on visualization history and the popularity of the content, among other information. Users can also actively search for videos using keywords or browsing channels. A user can interact with a video by watching it, liking, sharing with others, and/or publicly commenting, all of which are important social media features [22,23,24].

Previous research mentions that YouTube^®^ is one of many people’s favourite ways to learn how to cook [20]. Understandably, when compared to just text and images, recipes shared through video technology can favour user engagement, increase the motivation to cook, and reduce the perception of time, skills, and cost barriers [25]. Video recipes also potentially assist with the development of new skills, increase the pleasure of cooking, provide real-time assurance during the cooking process, help people remember the steps, and improve the understanding of the process [26]. In Brazil, YouTube^®^ is the most popular social media platform among individuals aged between 16 and 64 years [16].

Accessing the Internet to search for recipes, learn how to cook, and develop cooking skills is recommended by the Dietary Guidelines for the Brazilian Population [3], but the healthiness of recipes obviously depends on the ingredients and preparation methods employed [11]. In this sense, exploring the sources of knowledge and inspiration to cook is as key as getting people to cook more often. As tools that guide the preparation of dishes [27], culinary recipes can potentially promote health if aligned with recommendations for healthy eating, expanding and encouraging individuals’ decision-making autonomy regarding the adoption of healthy eating practices [2,18]. However, in the context of social media, content can be produced and shared by anyone, including lay people not qualified to give nutritional advice or create content that promotes healthy eating.

Previous studies assessed the healthiness of Internet recipes on websites and blogs (which are not social media), and concluded that users tend to interact more often with the least healthy recipes [28]. Authors concluded that even recipes tagged as ‘healthy’ are often quite unhealthy [28,29]. We identified only one paper on the healthiness of culinary recipes on social media, which used Pinterest^®^ as a data source [30]. The paper reported that recipes using seafood or vegetables as main ingredients had fewer calories, sodium, sugar, and cholesterol than meat- or poultry-based recipes. However, the study’s sample was small due to the adoption of many exclusion criteria [30]. No research investigating the healthiness of culinary recipes shared on other social media was found.

To address this gap, this descriptive and exploratory study analyses the healthiness of culinary recipes shared on popular YouTube^®^ cooking channels from Brazil, using both national dietary guidelines as references. We adopted complementary approaches to assess recipes’ healthiness, the first being the analysis of recipes’ ingredients according to the extension and purpose of industrial processing, an important and widely used approach to categorize foods. Subsequently, a specially designed qualitative framework was used to characterize recipes according to cooking method, and by the presence of healthy or unhealthy ingredients. We believe this study has the potential to inform the design of public health initiatives that guide individuals and inform dietitians on how to select and critically evaluate sources of cooking information, and improve the quality of homecooked meals.

## 2. Materials and Methods

Considering the scarcity of literature on the research topic, a pilot study was carried out to inform the data collection protocol, which included aspects of various channels’ eligibility criteria and database layout, introduced different video characterization variables, validated recipes’ assessment method, and determined the data collection period, taking into account the temporal feasibility of the study and the amount of content to be analysed [31].

### 2.1. Selection of YouTube^®^ Cooking Channels

**Figure 1:**
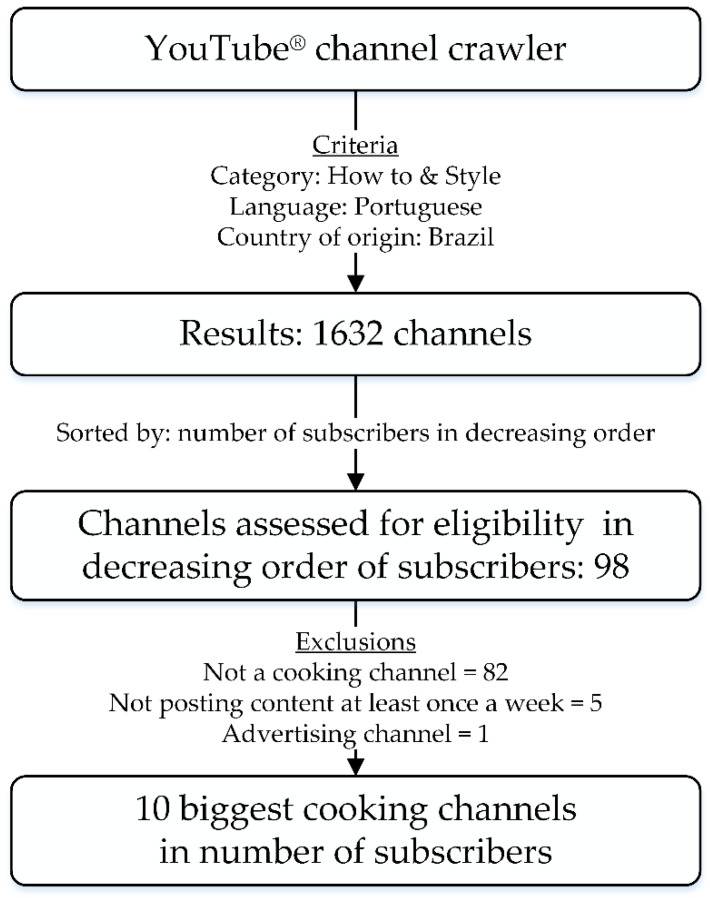
Brazilian YouTube^®^ Cooking channels’ selection flowchart, February 2020.

Cooking channels were purposely selected by taking into consideration that popularity (number of subscribers) can promote a greater reach and be a proxy for users’ preference. Channels were selected according to the number of subscribers in February 2020 using The YouTube Channel Crawler page (https://www.channelcrawler.com/, accessed on 10 February 2020), which classifies channels according to criteria established by the researcher (in this study: category, language, country of origin, and number of subscribers). During the pilot study, it was observed that cooking channels belonged to the ‘How to and Style’ category on YouTube^®^, thus, all channels of the platform within that category were accessed in decreasing order of subscribers to identify which ones best fit the eligibility criteria. The ten biggest channels which (1) presented audio-visual content in Portuguese and were Brazilian based; (2) were a cooking channel; (3) posted culinary videos at least once a week; and (4) were not an advertising channel or reproduced television cooking programs were selected (Figure 1).

With the aim of having a high number of videos to be analysed during data collection, it was established that channels that posted videos less than once a week would not be included. The pilot study also revealed that some channels which were among the most popular in terms of number of subscribers had suddenly stopped producing content in the weeks preceding the selection of channels. They were not included to avoid the possibility of not having enough content to analyse in the following months. Another reason for adopting this criterion was to try to standardize the number of videos per channel. Novelty was another important factor, as channels need not only to attract, but also maintain users’ interest and engagement with content [23].

Eighty-two channels were excluded from the sample because they were not cooking channels, five were excluded because they did not post videos with the desired frequency, and one was excluded for being an advertising channel.

The included channels were mostly presented by women (*n* = 7), two by men, and one by a couple; none of them were popularly known chefs or food celebrities. Subscribers ranged from 514 thousand to 4.25 million; channels’ time of existence ranged from 4 to 9 years, and posting frequency varied from 2 to 7 videos per week.

### 2.2. Selection of Recipes

A sample of 823 recipes presented in 755 videos (104 h and 21 min in total) posted during a six-month period (from February to August 2020) on ten different cooking channels was selected. Considering that this is a recent field of study and there is no specific recommendation in the literature for how long data collection on YouTube^®^ should take place, the pilot study also informed the choice of an appropriate data collection period. With the pilot study, we were able to project that a 6-month data collection period would capture a high number of videos from each channel, carefully accounting for at least three seasons of the year. At the same time, the amount of content collected would meet the temporal and operational feasibility criteria of the study.

All videos with recipes posted within the period were watched in full (first author) to determine if they contained all the ingredients needed, as well as the preparation method. A total of 106 videos were excluded from analysis because they did not meet the eligibility criteria: (1) were recorded live transmissions (*n* = 35), (2) presented a festive recipe (Easter *n* = 20; Mother’s day or father’s day *n* = 6; Valentine’s day = 4; June festivities in Brazil *n* = 12; Channel’s subscribers milestone celebration *n* = 4; total *n* = 46), (3) were sponsored by the food industry (*n* = 9), (4) were a repost (*n* = 7), (5) presented recipes linked to the COVID-19 pandemic (with connotations of treatment for the virus, for improving immunity or with tips for food sales during the period of social isolation; *n* = 9).

Reasons for not including recorded live transmissions were: (1) during the pilot study, we observed that those kinds of videos were usually presented as ‘extra’ content and were produced by only four of the ten channels. They were not included for the sake of standardization (type and number of videos per channel). (2) ‘Live’ transmissions lasted more than one hour each, as the recipe-related content was diluted among various other content during the video. This affected both the practical relevance of the recipe and the temporal feasibility of the research.

### 2.3. Data Collection

Weekly, from February to August 2020, each selected channel was accessed via computer and all videos posted during the previous week were registered. A database in Microsoft Excel 2016^®^ was created to include the following information for each video: title, access link, ID provided by YouTube^®^, video description, date of posting, date of access, duration in seconds, number of likes, dislikes, and views. To obtain the number of comments posted by users in each video, a command line application was developed in Python 3.0 (third author). Using the public and free Google Data application programming interface (API) service^®^ as the data source, the application generated automated reports from the video ID and the period determined by the researcher (freely available at https://bitbucket.org/amcamargo/healthy-recipe-youtube-br.git, last accessed on 11 August 2020).

Next, the first author watched each video to register the ingredients and the cooking method in the database. If further details about ingredients were needed, the researcher consulted the recipe’s ingredient list provided in the video description, or, in case of industrialized products, the packaging, when information was clearly visible on screen. Steps or ingredients mentioned by the youtuber as ‘optional’ and not shown in the video were not assessed.

### 2.4. Data Analysis

#### 2.4.1. Videos’ Characteristics

Variables assessed to characterize the videos were duration in minutes, day of the week of posting, and interaction measures including popularity as daily views in the first week, approval as daily likes and dislikes in the first week, direct interaction of users with content through daily comments in the first week, and total comments in the first week and in the first month after the video was posted.

To classify the recipe into a category (e.g., salad, pudding, etc.) a content analysis was carried out based on the video’s title, description, and list of ingredients used. This analysis was manually organized in Microsoft Excel 2016^®^ by determining the degree of similarity of the words and phrases used and the characteristics of the recipes, starting at coding recipes’ names in videos’ titles (first author). After coding, data was categorized until strong or terminal categories appeared [32].

#### 2.4.2. Recipes’ Healthiness

Recipes had their ingredients classified according to the extension and purpose of industrial processing, as unprocessed/minimally processed (U/MP), processed culinary ingredient (PCI), processed (P), or ultra-processed (UP) (first author) [3,4,33]. Ingredients that did not have their preparation described in the recipe but are available for purchase as an industrialized version were classified as P or UP (e.g., sweetened condensed milk, mayonnaise), according to the predominant characteristic of products available in Brazilian retail outlets. Whenever agreement about the extension and purpose of industrial processing was not achieved, a conservative criterion was applied, meaning that a lower extension of processing was adopted for the ingredient [34]. Ingredients used twice in the same recipe counted as one (e.g., sugar used in a cake’s batter and icing).

Subsequently, the Qualitative Framework for the Assessment of Culinary Recipes’ Healthiness [31] was applied to evaluate recipes’ cooking methods and presence of key healthy and unhealthy ingredients (first author). The framework was specifically developed and validated to assess culinary recipes’ healthiness, and was based on recommendations for healthy eating retrieved from both Dietary Guidelines for the Brazilian Population [2,3].

#### 2.4.3. Data Treatment

To ensure data quality control, the second author independently analysed 10% of the recipes from the dataset. Weighted kappa of agreement between raters for the assessment of ingredients’ extension and purpose of industrial processing was 0.96, and ranged between 0.90 and 1.00 (kappa and weighted kappa) for the application of the Qualitative Framework for the Assessment of Culinary Recipes’ Healthiness, indicating almost perfect agreement in both analyses [35]. Content analysis for the categorization of recipes was firstly discussed between the first two authors, and divergences were resolved with the participation of the last author.

#### 2.4.4. Statistical Analysis

Qualitative dichotomous and polytomous variables are presented in absolute and relative frequencies. Quantitative variables are presented as median and interquartile range (IQR), considering the non-normality in data distribution when assessed by Shapiro–Wilk test, histogram, kurtosis value, and mean/median proximity.

Variables of videos’ characteristics and recipes’ healthiness among the categories of recipes were compared. Also, as data collection took place mostly during a social distancing period due to the COVID-19 pandemic, when searches for recipes online increased [36], we also checked for differences in videos’ interaction measures (popularity, approval, interaction through comments), and recipes’ healthiness in the periods preceding (*n* = 141) vs. during social isolation (*n* = 614) (which, in Brazil, started around 15 March). Mann–Whitney and Kruskall–Wallis tests were used for quantitative variables. For qualitative variables, Pearson’s chi-square test was employed. Stata 13.0^®^ (StataCorp LLC, College Station, TX, USA) was used for analysis and a post-hoc power analysis was applied on G*power 3.1.9.2 whenever necessary, considering a two-tailed test. An alpha of 0.05 was established as the significance level for all analyses.

## 3. Results

### 3.1. Videos’ Characteristics

The videos’ durations ranged from 45 s to 27.33 min (*n* = 755). The number of daily likes in the first week was superior to daily dislikes. The option of liking or disliking a video was not enabled by the youtubers for all videos (only *n* = 611), therefore, even if users wanted to give a particular video a thumbs-up or down, they could not. Direct interaction through comments was concentrated in the first week after the videos were posted, as the median of total comments in the first month was close to the median in the first week. Sunday was the day of the week with the lowest number of videos posted, nevertheless, the distribution of videos was similar among the other days (Table 1).

The only observed difference between videos collected in the period preceding vs. during social isolation was in the total of comments in the first week, which was higher during the social isolation period (median = 154, IQR = 64; 280) than before the pandemic (median = 140; IQR = 47; 234) (Mann–Whitney’s *p* = 0.04, power = 0.19).

More than two thirds of all recipes (68.1%) comprised preparations from only four categories, namely: meat or egg main dishes; cakes and baked goods; snacks and homemade fast foods; and puddings (Table 2). The sixteen different categories of recipes had comparable video characteristics (all Kruskall–Wallis *p* > 0.10; χ^2^ = 91.19, *p* = 0.445). The frequency of categories observed in the period preceding vs. during social isolation was statistically the same (χ^2^ = 18.25; *p* = 0.07).

### 3.2. Recipes’ Healthiness

Of the total 7814 ingredients analysed, the majority were U/MP (54.3%, *n* = 4242) and PCI (23.6%, *n* = 1844). Ingredients classified as P (8.6%, *n* = 676) and UP (13.5%, *n* = 1052) were less frequent. The categories of recipes differed in terms of the ingredients’ distinct extension and purpose of industrial processing (χ^2^ = 859.22; *p* < 0.001). As Table 2 shows, in many categories, less than half of the ingredients were U/MP, i.e., cakes and baked goods, snacks and homemade fast foods, puddings, breads, sweet and savoury spreads, and pâtés. The ten most frequent U/MP ingredients in the sample were, in decreasing order: water, eggs, onion, all-purpose flour, garlic, milk, black pepper, oregano, spring onions, and tomatoes. Some of the categories with the lowest frequency of U/MP foods also had the highest frequencies of UP foods in the sample, i.e., puddings, cakes and baked goods, snacks and homemade fast foods, sauces, sweet and savoury spreads, and pâtés. The ten most frequent UP ingredients in the sample were, in decreasing order: UHT cream, sweetened condensed milk, Brazilian cheese spread, margarine, ham, industrialized tomato sauce, spicy sausage, vanilla essence, industrialized seasoning mix, and semi-sweet chocolate. The frequency of ingredients with distinct extension and purpose of industrial processing observed in the period preceding vs. during social isolation was not statistically different (χ^2^ = 0.68; *p* = 0.877).

Application of the Qualitative Framework for the Assessment of Culinary Recipes’ Healthiness (Table 3) identified positive and negative aspects of the recipes. Positively, most recipes that mentioned some type of fat as an ingredient did not suggest the use of margarine (88.4%, *n* = 518). Mentions of tomato sauce with herbs (bottled or freshly made) were more frequent than exclusive mentions of white sauce with mayonnaise or cheese (69.6%, *n* = 131). Exclusive use of industrialized seasonings (1.5%, *n* = 7) and of frying as a cooking method (7.9%, *n* = 60) was also not frequently mentioned. On the other hand, the presence of whole cereals, breads and/or pasta, either exclusively or mixed with refined cereals was low in the recipes (7.1%, *n* = 34), as well as were the presence of fruits (13.7%, *n* = 111), legumes (4.5%, *n* = 37), and nuts and seeds (3.5%, *n* = 28). The categories that presented the most evenly distributed positive and negative criteria were types of meats, presence of foods with high sugar concentration, and presence of vegetables. All results from the framework analysis were statistically the same regarding the period of data collection (preceding vs. during social distancing; all 0.01 < χ^2^ > 4.73 and *p* > 0.07).

## 4. Discussion

This study analysed the healthiness of recipes shared on popular YouTube^®^ cooking channels from Brazil using the Dietary Guidelines for the Brazilian Populationas references. Recipes posted during a six-month period were retrieved and categorized into sixteen different groups. The most frequently posted recipes were of meat/egg-based main dishes; cakes/baked goods; snacks/homemade fast foods; and puddings. This means that recipes for salads and side dishes, which usually contain vegetables, fruits, and legumes, were shared less often than recipes with animal sources of protein, all-purpose flour, fats, and sugar as the main ingredients. This result is not favourable from a health standpoint, as individuals are possibly being led to prepare fewer recipes with fruits, vegetables, and legumes, which are linked to a lower risk of chronic non-communicable diseases, and are largely present in most healthy eating patterns [37,38]. Interestingly, the study by Trattner and Elsweiler (2017) identified different results—in their study, which evaluated content from a recipes’ website, the category ‘fruits and vegetables’ was much more prevalent than ‘main dishes,’ ‘meat and poultry,’ ‘desserts,’ and ‘salads.’ This disparity may be attributed to differences in the process of categorizing recipes, as in food blogs [29,39] and websites [28], recipes are usually pre-categorized, while we conducted our own categorization. Because YouTube^®^ is a multi-content platform not specifically focused on recipes, our recipe categories were qualitatively and inductively generated from recipes’ titles, descriptions, and ingredients. Additionally, several studies only assessed specific categories of recipes [28,29,30,39], since their aim was not to have an overall picture of what is shared.

Another possible explanation for the low prevalence of fruit- and vegetable-based recipes in our sample may be that content producers expect users to interact with the postings through comments and shares, as interaction is fundamental for a channel’s engagement and sustainability [23]. It has been reported by previous studies on a recipes’ website [28] and on Pinterest^®^ [30] that interaction is more frequent with posts of highly palatable recipes. In our study conducted on YouTube^®^, every culinary preparation had statistically equal measures of interaction (popularity, approval, and direct interaction through comments), possibly due to differences between the profiles of users from recipe websites [28] and even between different social media apps [30]. YouTube^®^, as a video platform, enables a kind of interaction that gives users a feeling of being connected not only to a video, but to a person who shares their beliefs and interests. This feature can promote a certain measure of social bonding in which people feel connected with one another and start following the channel for further communication. For user-created content such as the videos analysed, a sense of community is fundamental; so it is possible that subscribers give the same attention to a recipe, regardless of whether it is a salad or a cake, in order to provide support through constancy of viewership and interaction [21]. The reasoning behind youtubers’ choices of categories of recipes for cooking videos, the channels’ features that promote connection with users, as well as subscribers’ motivations for interaction with content, deserve to be further explored in future research. Nevertheless, health professionals should be aware that, in order to expose individuals to more recipes based on vegetables, fruits, and legumes (such as salads and side dishes), active searching is preferable to just following content from popular cooking channels. Nutritionists and other health professionals can also search for cooking channels whose content is more in line with the healthy eating recommendations of national guidelines to suggest to patients.

Recipes’ ingredients were mainly U/MP foods and PCI, so one can argue that from a wide perspective, the recipes could lead individuals to cook recipes that are aligned with the recommendations of the Dietary Guidelines for the Brazilian Population [3]. Nevertheless, this is not true when different categories of culinary recipes are considered. Some categories of recipes had lower frequencies of U/MP foods as ingredients, and a few of them had, in addition to this, higher frequencies of the UP foods in the sample (more than 10%)—i.e., puddings, cakes and baked goods, snacks and homemade fast foods, sauces, sweet and savoury spreads, and pâtés. This result is cause for concern, as some of these were among the most frequently posted recipes. To cook healthily, the Dietary Guidelines for the Brazilian Population recommends the avoidance of UP foods [3], as high consumption of UP foods has been associated with chronic non-communicable diseases and all-cause mortality [5,6,7]. UP food consumption has been associated with a poor dietary intake (excess calories from free sugars and unhealthy saturated fats, poor in fibre, and an intake of many micronutrients) [40]. Additionally, recent research shows that the majority of the associations between UP food consumption, obesity, and health-related outcomes can be attributed to UP foods on their own, regardless of diet quality or pattern [41].

The presence of UP ingredients in the recipes may be explained by their convenience appeal [4,33]. It is rather common for UP foods to replace U/MP foods in recipes (e.g., sausage vs. U/MP meat seasoned with spices and herbs). Generations have learned to cook using recipes combining UP and U/MP foods through teaching investments by the food industry (leaflets, books, courses, recipes on packaging) [42]. Nowadays, the ongoing increase of options of UP foods and of social media marketing play an important incentivizing role [43]. We observed with our framework analysis (Table 3) that the mixed use of industrialized seasonings with fresh or dried herbs and spices was frequent, indicating an attachment to this type of UP product. To mitigate this effect, strategies involving the promotion of healthy eating through cooking (such as workshops, intervention programs, creation of content for social media, health professionals’ advice, etc.) need to consider that people must be taught how to identify UP foods so they can choose recipes in which they are not included. People must also be taught how to substitute UP foods for healthier ingredients, so they can use U/MP foods practically when cooking. For instance, instead of relying on UP foods as seasoning in puddings, snacks, and homemade fast foods as observed in this sample of recipes, one can substitute such ingredients for fruit zest and juices, fresh or dried herbs, and spices. Another valuable strategy is to rescue and promote the sharing of traditional recipes that do not contain UP foods as ingredients.

While the majority of recipes were healthy with respect to avoiding the use of margarine, avoiding frying, and opting for sauces with lower fat content, other aspects such as incorporating whole cereals, fruits, legumes, nuts, and seeds in preparation were not frequently present. Considering that social media platforms such as YouTube^®^ reach a wide audience, this finding reinforces the need to not only encourage people to look for recipes online [3], but also to teach them how to choose or adapt these recipes by evaluating their healthiness. One strategy is to use the same medium to do this, as video technology can help individuals overcome barriers to cook and incorporate healthier foods in recipes [44], while reducing the perception of barriers to cook with vegetables [25]. We are aware that, for some recipes, whole cereals, fruits, legumes, nuts, and seeds may not be all traditionally present (e.g., a basic homemade bread), but different ‘improved’ versions of recipes can be proposed and shared. As a matter of fact, many channels assessed in this study adapted recipes to keep producing new content weekly. As a practical implication, we argue that many categories of recipes can be adapted to become healthier—for examples and suggestions, see [31]. Members of academia, health professionals, and social media content creators can also work together and establish partnerships to promote healthier content on the Internet.

### Limitations and Strong Points

The adoption of a conservative criterion for classifying ingredients by the extension and purpose of industrial processing may have led to an underestimation of the number of UP ingredients. Nevertheless, this approach mirrors how information reaches users—they also do not necessarily have access to information on labels when watching videos.

Data collection took place during months of social isolation due to the COVID-19 pandemic, when searches for recipes online increased [36]. This was handled by avoiding the inclusion of videos linked to the COVID-19 pandemic in the sample. Our post-hoc analysis found an underpowered difference in the number of comments in the first week after videos were posted (power = 0.19) [45]; no change in the categories of recipes shared, nor in their healthiness compared to videos from before the pandemic.

YouTube^®^ channels’ popularity oscillates constantly. To handle this, we repeated the channel selection step at the end of data collection, and verified that they remained as the ten most popular in the period, despite some outperforming others in the number of subscribers.

As the number of views is validated by YouTube^®^’s own algorithms, a view might not indicate a user who has watched the content in its entirety. Content approval (likes and dislikes) also does not indicate whether or not an individual fully watched the content before giving a positive or negative rating. Although views made by computer programs rather than by humans are not counted [46], those interaction measures should be cautiously interpreted [47].

In the context of television cooking shows, some researchers argue that the consumption of this content is unlikely to impact habitual dietary intake, because entertainment and leisure are the main reasons people watch those programs [48,49]. Notwithstanding, social media, through its networked nature, provides an additional layer of complexity not experienced by those earlier media scholars [47]. Through observation, people indeed acquire behaviours, knowledge, values, and skills, including those related to cooking [50].

We understand that it is not possible within the confines of the present study to account for variations in the reproduction of recipes at home, such as instances when people do not follow all the steps, or when ingredients are exchanged, which may result in a different assessment of their healthiness.

As positive points, we highlight the investigation of culinary recipes posted in the most used social media in Brazil, by adults [16]; the rigorous quality control; the long period of data collection throughout three seasons of the year, and, therefore, the large sample size. Recipes were also very diverse in terms of categories, video duration, and days of posting, probably reaching different types of audiences. Additionally, collecting measures of interaction (views, comments, likes, and dislikes) reinforces the wide reach that this type of content has. Finally, using a validated framework for the assessment of recipes’ healthiness, we were able to deliver a more specific picture of the research problem.

## 5. Conclusions

This study provides a comprehensive overview of the healthiness of culinary recipes shared on a social media platform, one of the favoured avenues for the search of cooking-related content. On a professional practice and health promotion note, although it is praiseworthy that people are cooking and sharing their knowledge on platforms such as YouTube^®^, users and subscribers to popular cooking channels should be aware that most recipes are based on ingredients such as meats, eggs, all-purpose flour, fats and sugar, and only a few have whole cereals, fruits, legumes, nuts, and seeds. Recipes for puddings, cakes and baked goods, snacks and homemade fast foods, sauces, sweet and savoury spreads, and pâtés had, in addition to low numbers of U/MP food ingredients, higher numbers of UP foods as ingredients. Our findings can inform health professionals and policymakers on how to promote healthier culinary recipes, how to interact with content creators, and how to advise individuals about the quality of the recipes shared on YouTube^®^ videos, and hence, can help them choose healthier recipes or teach them how to modify the recipes into healthier versions. Future research exploring how users from different populational groups interact with culinary content on distinct social media platforms will be relevant for advancing this field of study.

## Figures and Tables

**Table 1:** Videos’ characterization variables (*n* = 755).

Variable	Median (IQR)
Duration (minutes)	7.8 (5.1; 10.8)
**Popularity**	
Daily views in the first week (*n*)	5194 (2094; 10,827)
**Approval**	
Daily likes in the first week (*n*)	995 (322; 1805) ^1^
Daily dislikes in the first week (*n*)	12 (3; 23) ^1^
**Direct interaction of users**	
Daily comments in the first week (*n*)	37 (14; 68)
Total comments in the first week (*n*)	150 (61; 272)
Total comments in the first month (*n*)	161 (67; 291)
**Day of posting**	**% (*n*)**
Monday	18 (136)
Tuesday	14 (103)
Wednesday	18 (138)
Thursday	16 (118)
Friday	15 (113)
Saturday	13 (97)
Sunday	6 (50)

Footnote: ^1^ *n* = 611 videos.

**Table 2:** Recipes’ healthiness according to ingredients’ extension and purpose of industrial processing.

Recipes’ Categories	Examples	Recipes % (*n*)	Ingredients Median (IQR)	Ingredients Distribution according to the Extension and Purpose of Industrial Processing			
				U/MP % (*n*)	PCI % (*n*)	P % (*n*)	UP % (*n*)
Meat or egg main dishes	Stroganoff, meat stew, omelette, chicken lasagne, one pan pepperoni pasta	22.8 (185)	12.0 (9.0; 15.0)	63.6 (1428)	16.1 (361)	9.1 (205)	11.2 (251)
Cakes and baked goods	Banana cake, chocolate cake, pies, biscuits, Brazilian cornbread, pancakes	18.2 (148)	8.0 (7.0; 10.0)	40.8 (530)	37.8 (491)	5.5 (72)	15.9 (206)
Snacks and homemade fast foods	Fried snacks, hotdog, pizza, sandwiches, *pão de queijo* ^1^, sweet popcorn	15.1 (123)	11.0 (7.0; 13.0)	49.6 (624)	23.5 (295)	13.4 (168)	13.9 (171)
Puddings	Mousses, sweetened condensed milk trifles, ice cream, rice pudding	12.1 (98)	6.0 (5.0; 7.0)	37.2 (225)	21.5 (130)	4.6 (28)	36.7 (222)
Side dishes	Cooked rice, *farofa* ^2^, cooked beans, roasted potatoes	9.2 (75)	10.0 (7.0; 12.0)	61.9 (440)	19.8 (141)	9.0 (64)	9.3 (66)
Breads	Basic homemade bread, homemade sliced bread, whole wheat bread, onion bread, aussie bread	5.0 (41)	8.0 (6.0; 10.0)	45.7 (148)	46.6 (151)	2.5 (8)	5.2 (17)
Savoury cakes and pies	Vegetable and cheese pie, sardine pie, quiche	4.8 (39)	14.0 (12.0; 18.0)	55.3 (306)	20.1 (111)	14.6 (81)	10.0 (55)
Salads	Raw vegetables with legumes salad, sautéed vegetables	2.6 (21)	9.0 (6.0; 13.0)	70.4 (143)	19.7 (40)	4.4 (9)	5.4 (11)
Soups and creams	Vegetables and/or chicken soups	2.2 (18)	13.0 (12.0; 14.0)	74.4 (169)	13.7 (31)	5.7 (13)	6.2 (14)
Appetizers	Onion toast, French fries, rice balls, fried beans	2.1 (17)	9.0 (6.0; 10.0)	61.8 (84)	25.0 (34)	8.1 (11)	5.1 (7)
Non-alcoholic beverages	Creamy coffee, hot-chocolate, juices	2.0 (16)	4.0 (4.0; 4.0)	63.8 (44)	17.4 (12)	5.8 (4)	13.0 (9)
Homemade ingredients	Butter, stock, homemade seasoning mix, pastry dough	1.3 (11)	5.0 (2.0; 9.0)	62.5 (40)	26.6 (17)	6.2 (4)	4.7 (3)
Savoury spreads and pâtés	Cheese pâté, dried tomato pâté, olive pâté	1.0 (8)	4.5 (4.0; 6.5)	45.8 (27)	27.1 (16)	15.2 (9)	11.9 (7)
Sauces	Bechamel sauce, pepper sauce, yoghurt sauce, rosé sauce	0.7 (6)	6.0 (5.0; 8.0)	51.6 (16)	25.8 (8)	-	22.6 (7)
Preserves	Onion preserve, beans preserve	0.5 (4)	5.5 (2.0; 9.5)	69.6 (16)	30.4 (7)	-	-
Sweet spreads	Dulce de leche	0.4 (3)	2.0 (2.0; 3.0)	28.6 (2)	28.6 (2)	-	42.8 (3)
Total		100.0 (813)	9.0 (6.0; 12.0)	54.3 (4242)	23.6 (1844)	8.6 (676)	13.5 (1052)

Footnote: U/MP—unprocessed/minimally processed foods. PCI—processed culinary ingredients. P—processed foods. UP—ultra-processed foods. ^1^ Traditional Brazilian recipe of small cheese bread made of fermented tapioca flour. ^2^ Traditional Brazilian dish made of manioc flour fried in fat, which can be enriched with other ingredients.

**Table 3:** Recipes’ healthiness according to the Qualitative Framework for the Assessment of Culinary Recipes’ Healthiness.

Category	Description of Components	Criteria	% (*n*)
Foods with high starch content	Exclusive presence of whole cereals, breads and/or pasta	+	5.0 (24)
	Mixed presence of whole and refined cereals, breads and/or pasta	+	2.1 (10)
	Exclusive presence of refined cereals, breads and/or pasta	−	92.9 (446)
Fruits, vegetables and legumes	Presence of vegetables	+	43.3 (353)
	Absence of vegetables	−	56.6 (460)
	Presence of legumes	+	4.5 (37)
	Absence of legumes	−	95.5 (776)
	Presence of fresh, frozen or dried fruits	+	13.7 (111)
	Absence of fresh, frozen or dried fruits	−	86.3 (700)
Nuts and seeds	Presence of nuts and seeds	+	3.5 (28)
	Absence of nuts and seeds	−	96.5 (784)
Meats and eggs	Exclusive presence of lean cuts of meat, poultry cuts without skin, fish, seafood and/or eggs	+	32.9 (109)
	Mixed presence of lean cuts of meat, poultry cuts without skin, fish, seafood and/or eggs and non-lean cuts of meat, poultry cuts with skin and/or processed meats	+	19.6 (65)
	Exclusive presence of non-lean cuts of meat, poultry cuts with skin and/or processed meats	−	47.4 (157)
Fats	Exclusive use of vegetable oils, butter and/or lard in place of margarine	+	88.4 (518)
	Presence of margarine	−	11.6 (68)
Sauces	Exclusive presence of tomato sauce with herbs	+	52.1 (98)
	Mixed presence of tomato sauce with herbs and white sauce, with mayonnaise or cheese	+	17.5 (33)
	Exclusive presence of white sauce, with mayonnaise or cheese	−	30.3 (57)
Seasonings	Exclusive presence of olive oil, lemon and/or fresh or dried herbs	+	68.9 (333)
	Mixed presence of olive oil, lemon and/or fresh or dried herbs, and industrialized spices, sauces and/or broths	+	29.6 (143)
	Exclusive presence of industrialized spices, sauces and/or broths	−	1.5 (7)
Sugars	Presence of foods with high sugar concentration	−	41.8 (338)
	Absence of foods with high sugar concentration	+	58.2 (470)
Cooking method	Use of steam, cooking in water without or with little fat, stewing, roasting, broiling, sautéing	+	92.1 (696)
	Use of steam, cooking in water without or with little fat, stewing, roasting, broiling, sautéing—and/or frying	−	7.9 (60)

Footnote: Fruits, vegetables, and legumes; nuts and seeds, and sugars categories are mandatorily assessed in all recipes. The remaining categories are assessed only when applicable. Criteria: + and − indicate recommended and not recommended components for healthy recipes, respectively [31].

## Data Availability

Not applicable.
